# Kappa and lambda light chain mRNA in situ hybridization compared to flow cytometry and immunohistochemistry in B cell lymphomas

**DOI:** 10.1186/1746-1596-9-144

**Published:** 2014-07-21

**Authors:** Lisa M Rimsza, William A Day, Sarah McGinn, Anne Pedata, Yasodha Natkunam, Roger Warnke, James R Cook, Teresa Marafioti, Thomas M Grogan

**Affiliations:** 1 Department of Pathology, University of Arizona, Tucson, AZ, USA; 2 Ventana Medical Systems, Inc., Tucson, Arizona, USA; 3 Department of Pathology, Stanford University College of Medicine, Stanford, CA, USA; 4 Robert J. Tomsich Pathology and Laboratory Medicine Institute, Cleveland Clinic, Cleveland, OH, USA; 5 Department of Pathology, University College Hospital London, London, UK

**Keywords:** Clonality, B cell lymphoma, Kappa, Lambda, In situ hybridization, Light chains

## Abstract

**Background:**

Detection of B cell clonality is useful for assisting in the diagnosis of B cell lymphomas. Clonality assessment can be accomplished through evaluation of *KAPPA* and *LAMBDA* light chain expression. Currently, only slide based methods are available for the majority of patient biopsies and do not detect light chain protein or mRNA in many B-cell lymphomas. Herein we evaluated a new method, known as colorimetric *in situ* hybridization (CISH), with improved sensitivity and multiplexing capacity, for its usefulness in clonality detection in mature B cell malignancies.

**Methods:**

The *KAPPA* and *LAMBDA* ISH was performed on a Ventana Benchmark XT utilizing two color chromogenetic detection. The probes comprised 2 haptenated riboprobes each approximately 500 base pairs long directed against the conserved regions of either *KAPPA* or *LAMBDA* mRNA. The dual colors consisted of silver deposition (black) for *KAPPA* light chain and a novel (pink) chromogen for *LAMBDA* light chain. Following optimization, CISH allowed visualization of mRNA in benign B cells in reactive tissues including germinal center, mantle zone, and post-germinal center cells. We then identified 79 cases of B cell lymphoma with formalin-fixed paraffin-embedded (FFPE) biopsies including: follicular (36 cases), mantle cell (6 cases), marginal zone (12 cases), lymphoplasmacytic (6 cases), small lymphocytic (4 cases), and diffuse large B cell (15 cases), which were selected on the basis of either prior flow cytometry or immunohistochemistry (IHC) results to serve as the predicate, "gold standard," comparator.

**Results:**

39/79 (49.4%) cases were classified as *KAPPA* and 29/79 (36.7%) as *LAMBDA* light chain restricted; while 9/79 (11.3%) cases were classified as indeterminate. Of the 70 cases with *KAPPA* or *LAMBDA* light chain restricted CISH, 69/70 (98.6%) were concordant with the reference method, while 1/70 (1.4%) was discordant.

**Conclusions:**

Optimized CISH detected lower levels of mRNA than can be visualized with current slide based methods, making clonality assessment in FFPE biopsies possible for mature B cell neoplasms. In this preliminary study, CISH was highly accurate compared to flow cytometry or IHC. CISH offers the possibility of wider applicability of light chain ISH and is likely to become a useful diagnostic tool.

**Virtual Slides:**

The virtual slide(s) for this article can be found here: http://www.diagnosticpathology.diagnomx.eu/vs/1430491067123856

## Background

Detection of B cell clonality has long been a useful tool for identification of monoclonal B cell populations, which are potentially malignant, from polyclonal B cell populations that result during the normal immune response [1]. The copy number range of light chain mRNA and protein expressed by B cells is variable and depends on stage of differentiation. While naive and memory cells may have only 10-100 mRNA copies per cell, plasma cells may have approximately 100,000 mRNA copies per cell [1]. Therefore detection methods need to have a wide dynamic range to be suitable for application in different types of B cell malignancies.

**Figure 1:**
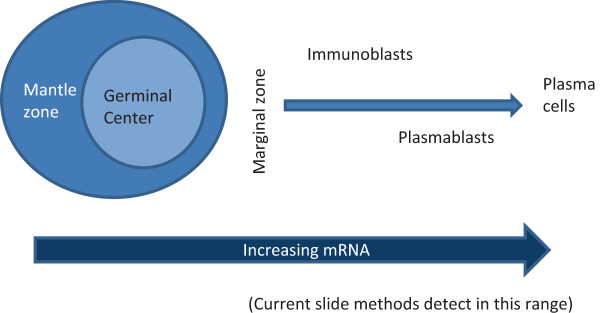
**Ig mRNA levels increase with B cell differentiation.** As B lymphocytes pass through stages of maturation from precursor B cells to naïve B cells to germinal center cells to post-germinal center cells then to plasma cells, the level of mRNA encoding immunoglobulin increases. Current slide-based methods are generally able to detect the mRNA levels found in the later stages of differentiation (generally in the post-germinal center stages).

Detection of B cell monoclonality in diagnostic pathology clinical laboratories can follow one of 4 basic methods. If fresh tissue is available, then disassociation of the lymphoid cells followed by immunofluoresence staining and flow cytometric analysis is common. This technique gives a level of sensitivity suitable for detecting the level of kappa or lambda protein found on the surface of all stages of B cell development [2]. If a permeabilization step is used in cell preparation prior to staining, then cytoplasmic light chain protein can be detected in plasma cells and lymphoplasmacytic cells [3]. While a highly successful method, the need for fresh unfixed tissue excludes many cases from analysis. If lymphoma is not suspected ahead of time or only a limited amount of biopsy material is obtained, then often only fixed tissues are available. In this common situation, immunohistochemical (IHC) detection methods of kappa and lambda light chain protein in formalin fixed, paraffin embedded tissues (FFPET) are widely used. However, these are somewhat limited by high background due to the presence of normal physiologic interstitial Ig and the low abundance of *KAPPA* and *LAMBDA* mRNA and protein on normal and malignant germinal center and pre-germinal center B cells [4]. Assessment of mRNA avoids the issue of interstitial Ig signal since mRNA is short-lived and intra-cytoplasmic. *In situ* hybridization (ISH) for mRNA is therefore used in many laboratories. However, current methods allow visualization of mRNA levels of B cells only in the later stages of differentiation and so do not have the dynamic range of flow cytometry (see Figure 1) [5,6] Finally, molecular methods aimed at amplification of *KAPPA* and *LAMBDA* light chain genes and examination for a predominant rearrangement motif are also used. These techniques usually performed in molecular laboratories, require longer time as nucleic acid extraction and amplification are needed and do not retain morphologic context i.e. exact visualization of the cell of interest [7]. Therefore, despite a variety of current methods, there are still many cases where assessment of B cell monoclonality is technically challenging, posing a limit to the diagnostic process.

In diagnostic pathology, ISH on histologic sections commonly starts with haptenated probes (labeled with either DNP (2,4-dinitorphenol) or DIG (digoxigenin), followed by an anti-hapten antibody, and an indirect biotin streptavidin system with either alkaline phosphatase or silver deposition for visualization [8;9]. Clinical examples common in diagnostic hematopathology include detection of *KAPPA* and *LAMBDA* mRNA, as discussed above, as well as Epstein Barr Virus Encoded RNAs (EBER). New probe design approaches eliminating any repeat segments, as well as the development of novel haptens and chromogens have opened the possibility for more specific staining with greater sensitivity and the detection of multiple probes per slide [8,9].

Given the advantages of ISH (suitability for fixed tissues, absence of background) as well as technological developments in the field, the current study sought to develop a new methodology known as dual color *in situ* hybridization (CISH) and to compare its performance in assessing B cell clonality to more commonly employed methods in a series of non-Hodgkin B cell lymphomas. Herein, we describe for the first time this new method and report initial promising results for future clinical application.

## Methods

### Tissues

Benign tissues included 2 tonsils removed for tonsillitis and 1 with reactive paracortical hyperplasia. B cell non-Hodgkin lymphomas (B-NHL) cases were assembled as follows: Stanford University submitted 19 cases with flow cytometry results, Cleveland Clinic submitted 18 cases with flow cytometry results, the University of Arizona submitted 27 cases with flow cytometry results, and the University College of London submitted 15 cases with IHC results. The case criteria included a diagnosis of mature B-cell non-Hodgkin lymphoma, remaining tumor in the paraffin block, and documented monotypic light chain pattern by flow or IHC. Each site performed flow cytometry according to their own clinical laboratory standards and so was not standardized across the case series. Decalcified samples from bone marrow were not included since the method is not yet optimized for that setting. This project was conducted under a human subjects research ethical approval from the University of Arizona Institutional Review Board (protocol # 0500000226) according to the Declaration of Helsinki.

### Probe synthesis and formulation

Novel *KAPPA* and *LAMBDA* anti-sense probes, complementary to mRNA encoding each light chain constant region, were chemically labeled with different haptens using Mirus linker arms as directed by the manufacturer (Mirus Bio LLC, Madison, WI). Twenty-five nanograms of each probe was suspended in one mL of Ribohybe™ (cat. 760-104, Ventana Medical Systems) solution and placed into a dispenser.

### *RNA* in situ *hybridization and tyramide-chromogen detection*

**Figure 2:**
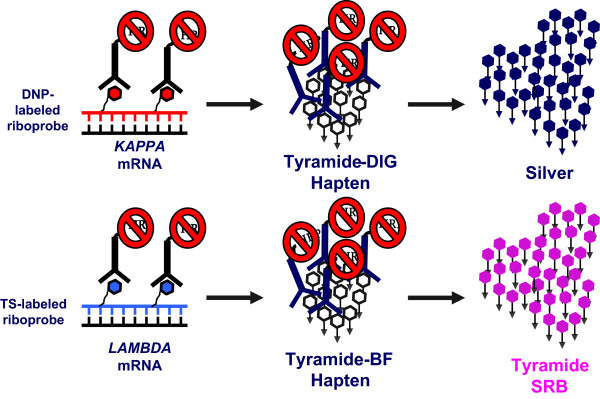
**2-Color RNA Detection Methodology.** A cocktail of antisense hapten-labeled riboprobes, targeting the constant region of either *KAPPA* or *LAMBDA* mRNA, was hybridized to de-paraffinized protease treated tissue samples. Following stringency washes the haptens were sequentially detected using a cognate anti-hapten monoclonal antibody conjugated to HRP which catalyzed deposition of a tyramide-hapten conjugate. Following inactivation of the HRP conjugate, each amplified hapten was then sequentially detected using a second anti-hapten antibody-HRP conjugate which catalyzed silver or pink chromogen deposition.

All steps were executed using a VENTANA Benchmark XT automated staining instrument (Ventana Medical Systems). Formalin-fixed, paraffin-embedded tissue samples mounted on Superfrost slides were de-paraffined and antigen retrieved using CC1 reagent and protease 3 (cat. 760-2020, Ventana Medical Systems). Following retrieval, cocktailed hapten-labeled *KAPPA* and *LAMBDA* experimental anti-sense strand probes were dispensed onto a slide, denatured at 80°C for 8 min, and hybridized at 65°C for 6 hrs. Following hybridization, slides were washed 3 times using 0.1× SSC at 75°C for 8 min. to remove non-specifically hybridized probe. Brightfield detection of each bound probe required a two-tiered sequential amplification strategy as follows. Endogenous tissue peroxidase activity was inactivated using PO inhibitor (cat. 760-4143, Ventana Medical Systems). The *KAPPA* probe was detected using an anti-*KAPPA* probe hapten monoclonal antibody conjugated to horseradish peroxidase/HRP to catalyze hapten amplification using a tyramide-hapten conjugate and TSA-H_2_O_2_ reagent (cat. 760-4141, Ventana Medical Systems). The procedure was repeated to detect the labeled *LAMBDA* probe using tyramide-mediated amplification of the second hapten. Control studies demonstrated that three successive applications of PO inhibitor were required prior to *LAMBDA* signal amplification to inactivate the HRP-conjugates used to amplify the *KAPPA* probe signal; omission of the inactivation step resulted in co-localization of signals and non-specific mRNA signals. Chromogenic detection of the *LAMBDA* then *KAPPA* associated haptens was accomplished sequentially using a similar amplification strategy which included in order three applications of PO inhibitor, application of a cognate anti-hapten monoclonal antibody and application of a tyramide-sulforhodamine B chromogen conjugate (for *LAMBDA* mRNA) before using a similar strategy to deposit the silver chromogen (for *KAPPA* mRNA) (See Figure 2). Tissue nuclei were stained using Hematoxylin; slides were then dehydrated using gradient alcohols and coverslipped. Cognate cocktailed sense strand probes were used as negative controls to determine background resulting from non-specific probe interactions.

### Slide interpretation

The slides were interpreted by 3 pathologists (SM, TG, LR) without knowledge of the flow cytometry or IHC results. Each pathologist initially read all slides independently without access to each other’s interpretations. Results between pathologists were then compared, followed by discrepancy resolution by joint microscopic review to achieve consensus. After a consensus interpretation was achieved, the CISH results were unblinded and compared to the flow cytometry and IHC results.

Dual color CISH for kappa/lambda mRNA is a new technology with results not before visualized. Positive staining was therefore carefully defined as demonstrating a partial to full ring of punctuate cytoplasmic staining. The signals were small and delicate similar in size to iron particles identified in erythroid precursors using an Prussian Blue stain on bone marrow aspirate smears. In cells at the late stage of B cell differentiation such as plasma or plasmacytic cells, the staining was sometimes heavy enough to obscure the entire cell. Nucleolar staining was considered non-specific. Indeterminate staining was assigned when there was a lack of or minimal staining on the malignant B cells (no rings or partial rings) and the plasma cells in the sample were positively stained or absent. Failed staining was assigned when there was no or minimal staining on malignant B cells (no rings or partial rings) and the plasma cells, which are expected to be strongly stained, were present but unstained.

## Results and discussion

**Figure 3:**
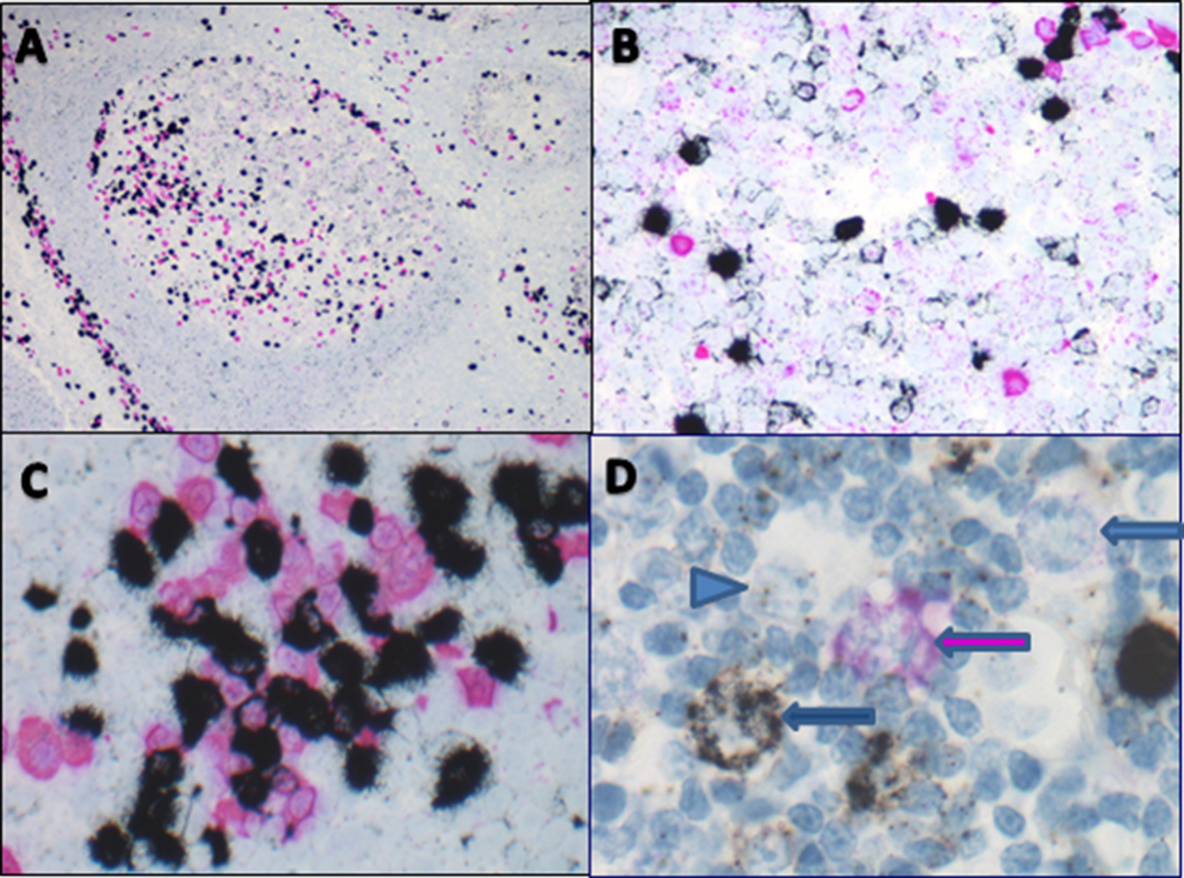
**Staining Pattern in Reactive Lymphoid Tissues. A**: Low power view of tonsil showing polyclonal staining within germinal center and mantle zone, and scattered paracortical B cells as well as intra-germinal center and paracortical plasma cells (40×). **B**: High power view of germinal center demonstrating polyclonal centrocytes, centroblasts, and a few plasma cells (500×). **C**: High power view of medullary sinuses with polyclonal plasma cells (600×). **D**: High power view of reactive paracortical hyperplasia showing *KAPPA* restricted B-immunoblast (black arrow), *LAMBDA* restricted B-immunoblast (pink arrow), negatively staining presumed T-immunoblast (blue arrow), and negatively staining histiocyte (blue arrowhead) (1000×).

Reactive tissues demonstrated a polyclonal pattern of staining including both kappa expressing (black labeled cells) and lambda expressing (pink labeled cells) (Figure 3). Within the germinal centers, centroblasts, centrocytes, and plasma cells stained in a polyclonal pattern. Polyclonal staining of the mantle zone lymphocytes could also be visualized. As expected, the plasma cells in the medullary sinuses were also polyclonal. In a lymph node with reactive paracortical hyperplasia, a mixture of *KAPPA* and *LAMBDA* expressing immunoblasts were identified as were immunoblasts which were negative for both *KAPPA* and *LAMBDA* as well as *KAPPA* and *LAMBDA* negative immunoblasts, which were presumed to be T-cell immunoblasts.

**Figure 4:**
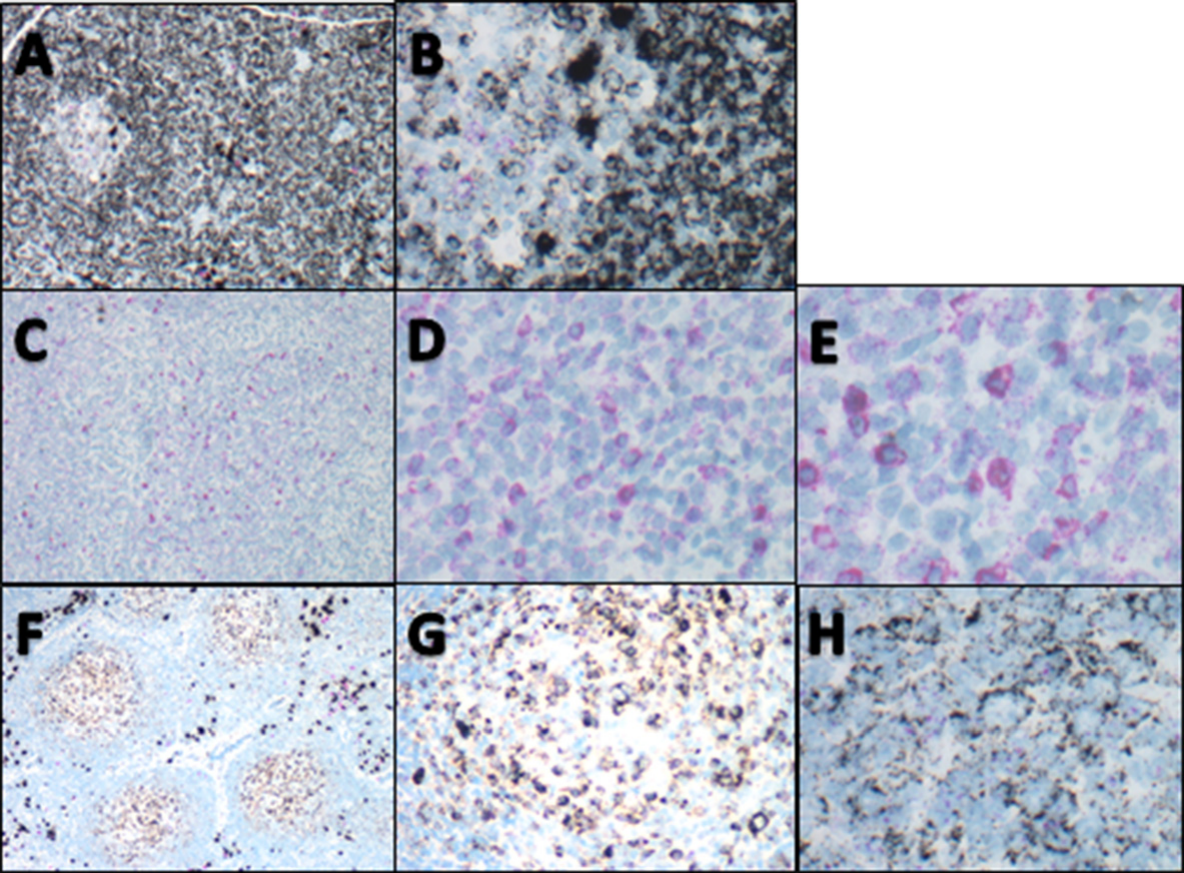
**Light chain restriction in B cell lymphomas. A-B**: CLL/SLL **(A)** residual germinal center on left with sheets of monoclonal *KAPPA* light chain restricted lymphoma cells on the right (100×); **(B)** higher power view of *KAPPA* light chain restricted cells (500×). **C-E**: DLBCL **(C)** diffuse sheets of *LAMBDA* light chain restricted cells (100×); **(D)** same case showing intermediate magnification of *LAMBDA* light chain restricted lymphoma cells (400×); **(E)** same case showing higher power view with centroblastic morphology and *LAMBDA* light chain restriction. Rather than an inherent difference in the chromogens, but rather due to the larger cell size and lower nuclear to cytoplasmic ratios in DLBCL as compared to SLL or FL, the cellular detail is easier to appreciate in **E** than in images **B** or **H** (600×). **F-H**: Follicular lymphoma **(F)** low power view showing neoplastic germinal centers with *KAPPA* light chain restriction and cuff of polyclonal plasma cells (100×); **(G)** same case at intermediate power showing *KAPPA* light chain restricted FL; **(H)** same case at higher power showing *KAPPA* light chain restriction of both small and large neoplastic cells (600×).

After determining that the dual CISH assay was able to detect *KAPPA* and *LAMBDA* mRNA in mature B cells of increasing differentiation stages as encountered in peripheral lymphoid tissues, we next collected cases of B-NHL known to arise from the various lymph node populations (germinal center-, mantle zone-, marginal zone-derived lymphomas). Benign precursor B cells found in bone marrow and lymphoblastic leukemia/lymphomas were not included. There were 79 total cases which met all inclusion criteria representing 34 cases of follicular lymphoma (FL), 7 cases of mantle cell lymphoma (MCL), 11 cases of marginal zone lymphoma (MZL), 6 cases of lymphoplasmacytic lymphoma (LPL), 15 cases of diffuse large B cell lymphoma (DLBCL), 4 cases of chronic lymphocytic leukemia/small lymphocytic lymphoma (CLL/SLL), and 2 cases of small B-cell non-Hodgkin lymphoma, not otherwise specified. Photomicrographs of representative cases are shown in Figure 4. As seen in Table 1, 70/79 cases (88.6%) were found to have either *KAPPA* (39/79, 49.4%) or *LAMBDA* (29/79, 36.7%) light chain restriction in the presence of appropriate residual plasma cell staining. The remaining cases were classified as indeterminate (9/79, 11.3%) using the criteria described in Methods. Of these 9 indeterminate cases, 5 lacked staining in B cells with positive plasma cell staining and 4 lacked staining in B cells without discernible plasma cells in the section (to serve as tissue positive control). Therefore 75/79 (95%) of cases were considered to have sufficient mRNA integrity to be reliably interpreted. Of the 70 cases with interpretable CISH, 69/70 (98.6%) were concordant with the reference method (either flow cytometry or IHC), while 1/70 (1.4%) was discordant (shown by diagnostic category in Table 2). The latter case was a follicular lymphoma that showed *KAPPA* restriction by CISH and *LAMBDA* by IHC. Further examination of the discrepant cases demonstrated that 6/34 FL (17.6%), 1/11 MZL (9%), and 2/15 DLBCL (13.3%) were deemed indeterminate by CISH but were found to be evaluable by flow cytometry or IHC. These cases are detailed in Table 3.

**Table 1:** Total cases: concordant, discordant, indeterminate

	**Cases with minimum study criteria**	**Cases with interpretable CISH (excluding indeterminate cases)**
**Total**	79	70/79 (88.6%)
**Indeterminate**	9/79 (11.3%)	–
**Concordant with flow or IHC**	69/79 (87.3%)	69/70 (98.6%)
**Discordant with flow or IHC**	1/79 (1.3%)	1/70 (1.4%)

**Table 2:** Concordance of evaluable cases by lymphoma diagnosis

**Diagnosis**	**Concordance**	**Discordance**
**Follicular**	27/28 (96.4%)	1/28 (3.6%)
**Mantle Cell**	7/7 (100%)	0/7 (0%)
**Marginal Zone**	10/10 (100%)	0/11 (0%)
**Lymphoplasmacytic**	6/6 (100%)	0/6 (0%)
**Diffuse Large B-cell**	13/13 (100%)	0/13 (0%)
**SLL/CLL**	4/4 (100%)	0/4 (0%)
**Small cell B-NHL, NOS**	2/2 (100%)	0/2 (0%)
**Total**	69/70 (98.6%)	1/70 (1.4%)

**Table 3:** Indeterminate vs evaluable by lymphoma diagnosis

**Diagnosis**	**Cases**	**Indeterminate**	**Evaluable**
**Follicular**	34	6/34 (17.6%)	28/34 (82.4%)
**Mantle cell**	7	0/7 (0%)	7/7 (100%)
**Marginal Zone**	11	1/11 (9%)	10/11 (91%)
**Lymphoplasmacytic**	6	0/6 (0%)	6/6 (100%)
**Diffuse Large B Cell**	15	2/15 (13.3%)	13/15 (86.7%)
**CLL/SLL**	4	0/4 (0%)	4/4 (100%)
**Small B Cell NHL, NOS**	2	0/2 (0%)	2/2 (100%)
**Total**	79	9/79 (11.3%)	70/79 (88.6%)

Interestingly, 2 cases which were negative for surface Ig by flow cytometry were submitted to the study. Surface Ig negativity is well recognized and considered a sign of monoclonality in the workup of B-NHL, and has been reported in a variety of B cell lymphomas including follicular lymphoma [10-12]. In this study, 2 FL were surface Ig negative by flow cytometry. Both of these cases had "indeterminate" CISH results. Of these, 1 case contained positively stained polyclonal plasma cells (a true SIg negative case by all methods), while the other case did not contain any discernable plasma cells to indicate that staining was or was not successful. This latter case could therefore be considered either a true negative (non-Ig expressing lymphoma) or perhaps a case with poorly preserved mRNA.

**Figure 5:**
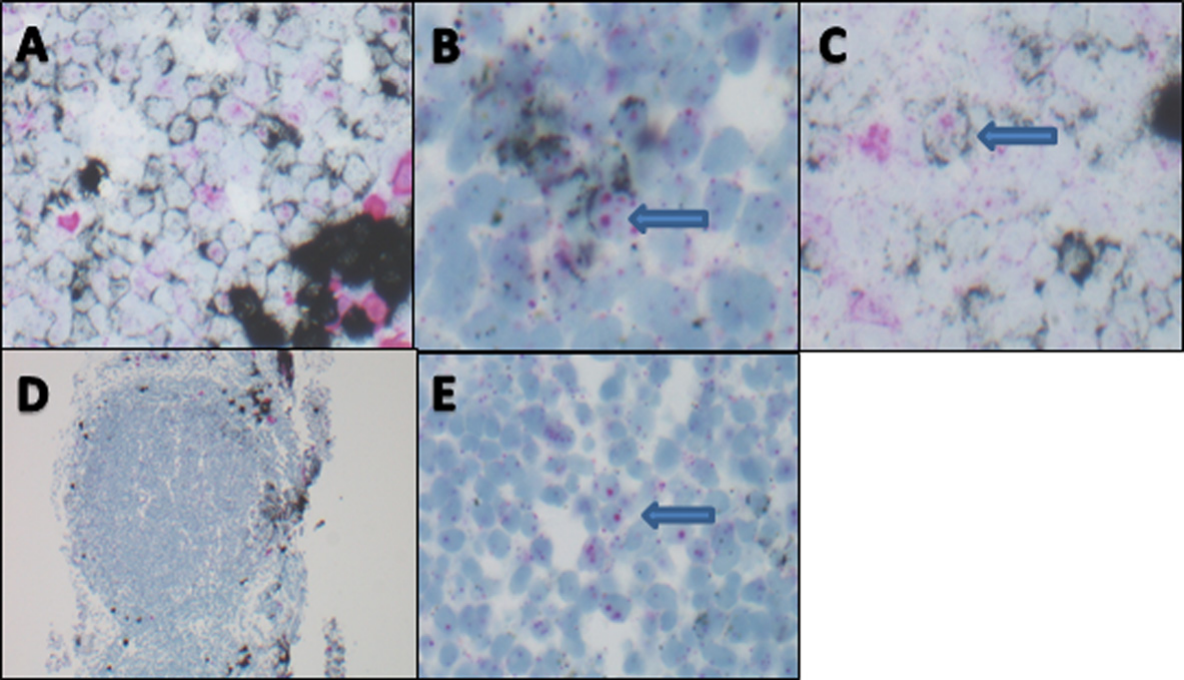
**Non-specific nucleolar staining with lambda probe. A-C**: Germinal center in a reactive tonsil. **(A)** polyclonal mixture of *KAPPA* and *LAMBDA* positive cells (400×); **(B&C)**: arrows point out *KAPPA* expressing cells as evidenced by black ring of cytoplasmic staining with non-specific pink nucleolar staining (600×). **D-E**: Follicular lymphoma. **(D)** low power view of FL case which was indeterminate for *KAPPA* and lambda due to expressing neither light chain (40×); **(E)**: high power view of same case demonstrating lack of cytoplasmic staining pattern for either *KAPPA* or *LAMBDA* light chain expression with non-specific pink nucleolar staining (600×).

A non-specific nucleolar staining was seen with the *LAMBDA* probe in 14 cases including all categories of lymphoma (a representative case is shown in Figure 5). Among the 14 cases, 6 showed *KAPPA* restriction, 5 *LAMBDA*, and 3 were classified as "indeterminate" by CISH. Because of the preservation of tissue morphology, this nucleolar staining was easily distinguished from the specific cytoplasmic pattern of *KAPPA* or *LAMBDA* mRNA staining.

When considering the diagnosis of lymphoma in patient biopsies, the monoclonality or polyclonality of the B cell populations is a critical component of the decision making process. While many methods can be used, the most common (flow cytometry) requires fresh tissue, or does not take into account morphologic context (PCR), or is insufficiently sensitive for the majority of B cell NHL (current IHC and ISH methods) [5,6]. In this study, we describe the feasibility and application of dual-color CISH technology for the evaluation of clonality of mature B cell populations in tissue sections. We demonstrate that with new, repeat-free probes and sensitive detection systems taking advantage of tyramide-induced deposition of chromogens, it is possible to detect not only *KAPPA* and *LAMBDA* mRNA levels typical of plasma cells and other post-germinal center B cells but even the very low level mRNA found in presumed naïve B cells in the mantle zones and germinal center B cells. This level of detection is then applicable to the lymphomas derived from most stages of B cell differentiation. The ability to automate the staining and then evaluate the results using light microscopy for slide evaluation holds the potential to easily integrate the CISH technique into routine diagnostic practice.

## Conclusions

In this study we describe a preliminary accuracy of 98% as compared to reference methods including flow cytometry and immunohistochemistry. There was only 1 case called *KAPPA* by CISH and *LAMBDA* by IHC, which is difficult to explain biologically. However, the explanation could be as simple as a technical or transcriptional error in the original report. Nevertheless, biological complexity does exist in the interpretation of the lambda CISH results due to the presence of non-specific nucleolar staining with the *LAMBDA* probe seen in several cases. Initially, this staining seemed to suggest conflicting results, particularly in the cases which had clear *KAPPA* mRNA cytoplasmic black colored staining while having apparent "*LAMBDA*" pink staining in the nucleoli of the same cells. However, a review of the literature revealed that this staining pattern may result from cross hybridization with the Ig lambda-like polypeptide 5 (*IGLL5*) gene on chromosome 22, which is located within the immunoglobulin lambda locus but does not require somatic rearrangement for expression, meaning that it can be expressed in non-Ig expressing, non-B, cells [13]. As recently demonstrated by Tubbs *et al*. cross-hybridization with the *LAMBDA* CISH probe occurs because while the first exon of *IGLL5* gene is unrelated to immunoglobulin variable genes, the second and third exons use the same loci as the Ig-lambda joining 1 and the Ig-lambda constant 1 gene segments [13,14]. Recognition of this non-specific pattern allowed interpretation of cases with pink nucleolar staining as well as black cytoplasmic staining as *KAPPA* restricted. In contrast, those with pink nucleolar staining without cytoplasmic staining were scored as "indeterminate". In at least 2 cases, "indeterminate" is the most accurate interpretation since by flow cytometry these were both considered surface Ig-negative cases.

Dual-color CISH allowed for detection of both *KAPPA* and *LAMBDA* mRNA on a single slide under a light microscope, making interpretation of B-cell clonality in tissue sections reliable and highly optimized in the context of tissue architecture and cellular compartments. This application should be of particular interest to pathologists engaged in the field of lymphoma diagnostics. The availability of additional novel haptens and chromogens, which can be paired with highly specific probes, allows for the possibility of additional multi-color combinations and innumerable future applications.

## Competing interests

Bill Day, Anne Pedata, and Tom Grogan are employees of Ventana Medical Systems, which supplied the probes and performed the staining for the project.

## Authors’ contributions

LR designed the study, interpreted results, and wrote the manuscript. WD designed the reagents, developed the techniques, and supervised experiments. SM identified cases, interpreted results, and wrote portions of the manuscript. AP designed the reagents, developed the techniques, and performed experiments. YN assisted in study design, supplied case materials, evaluated staining. RW conceived of the study, supplied case materials. JRC assisted in study design, supplied case materials. TM assisted in study design, supplied case materials. TMG conceived of the study, interpreted results, assembled data. All authors read and approved the final manuscript.
